# Help-seeking behavior of individuals with schizophrenia in the general population of Hunan, China

**DOI:** 10.1038/s41598-021-01819-w

**Published:** 2021-11-26

**Authors:** Jiawen Hu, Qiongjuan Zheng, Yun Zhang, Chunyu Liu, Xuefei Tian, Xuejun Liu, Dongxin Wang, Jing Ma

**Affiliations:** 1 School of Clinical Medicine, Hunan University of Chinese Medicine, Changsha, China; 2 Econd People’s Hospital of Hunan Province(Brain Hospital of Hunan Province), Changsha, China; 3 Northwest Minzu University, Lanzhou, China; 4 Department of Psychiatry, Department of Neuroscience & Physiology, SUNY Upstate Medical University, Syracuse, NY USA

**Keywords:** Health care, Medical research

## Abstract

This study aims to know the seeking help behavior of individuals with SZ (Schizophrenia) in Hunan province of China. Individuals (age > 15) with schizophrenia were recruited in the study after a two-stage diagnosis procedure (including questionnaire screening and face-to-face SCID interview by psychiatrists) in Hunan province. A self-designed questionnaire was used to investigate their help-seeking behavior. (1) Of the 367 participants, 68.9% (253/367) sought help; of those, 64.6% (n = 163) pursued professional psychiatric services and 30.8% (n = 78) pursued non-medical options (i.e., relatives, praying to Buddha) as the most common first choices. (2) Family history of mental disorders is significantly related to whether or not the individual with SZ seeks help, and the first choice of help is significantly related to education level. (P < 0.05). (3) Frequent reasons behind not seeking help include fear of stigmatization (72.9%), poor mental health literacy (64.5%), concerns over cost (50.6%), and limited access to medical services (47.0%). About one-third of the individuals do not seek help. Individuals with SZ tend to choose psychiatric hospitals and relatives as their first choice. Among the factors we investigated, family history of mental disorders is the most influential factor associated with help-seeking behavior. Individuals with more education tend to seek professional help first. The primary reasons for not seeking help include fear of stigmatization, lack of awareness about mental illness, concerns over cost, etc.

## Introduction

Schizophrenia (SZ) is a chronic, recurrent, and severe mental disorder prevailing in about 0.63% to 0.94% of the population (with an average annual rate of 0.81%) in china^1^. Most people with SZ benefit from antipsychotic treatment^2^ with early diagnosis is critical. Unfortunately, many individuals with SZ avoid getting help, estimates of 20% to 70% do not seek help worldwide as shown in the previous studies^3^. Many factors influence whether or not individuals with mental disorders seek help, such as gender, location of, annual income, type of medical insurance, marriage status, stigma, and other social and culture factors^4–7^. However, research focusing on whether or not individuals with SZ seek help is rare. Among those who do seek help, the first choice has recently attracted more attention. Early diagnosis of SZ can be very challenging because of patients’ hesitation or refusal in seeking help, especially professional help. For instance, in Saudi Arabia, Only 40% of individuals with SZ have had first contact with a health professional^8^. Shi and colleague’s study in China showed that 63% of individuals with SZ first sought help from non-psychiatric medical sources^9^. Many previous studies found that 16 to 76% of individuals with SZ seek help from relatives, friends, traditional/faith healers, and religion^10–16^. Moreover, the longer individuals have suffered from SZ, the lower their remission and recovery rates^17^. Experiencing SZ without treatment can mean more serious damage to social functioning, one’s own physical health, and the safety of others ^18,19^. Some research concludes that common choices for help include psychiatric hospitals^16^, general hospitals^20^, and psychotherapy^21^. Studies worldwide show that the most common first choices for individuals seeking help are general practitioners, traditional/faith healers, and psychiatrists^20,22^. Yalvac and colleague’s study (2017) show psychiatrists as the most common first choice for urban dwellers in Turkey^16^.

We hypothesize that the help-seeking ratio in china is low and people tend to ask for help firstly in such non-professional ways as traditional/faith healers, superstition, etc. This study aims to determine the help-seeking ratio and first choice of help for individuals with SZ living in the Hunan province of China.

## Methods

### Sampling procedures

The survey had two stages. The initial screening stage used a modified 12-item General Health Questionnaire (GHQ-12) and a severe mental disorders clue questionnaire^23,24^. The degree of risk (high/middle/low) was assessed according to the GHQ-12scoring.

The second stage involved diagnostic investigators, doctors from psychiatric hospitals who attended a unified training on SCID-I/P (Structured Clinical Interview for Diagnostic and Statistical Manual of Mental Disorders Fourth Edition, Text Revision Axis I Disorders, Research Version, Patient Edition, SCID-I/P). The investigators used SCID-I in all participants suspected of being at high risk and 30% of those suspected of having middle-risk suspected participants. For difficult diagnoses, two experts discussed the patient and then determined the diagnosis in consensus. A total of 539 individuals with schizophrenia (SZ) were eventually diagnosed within the second stage.

A self-administered questionnaire was used to collect the following information: whether or not individuals with SZ have sought help, the first choice for help, the main reason for not seeking help, whether or not the individual has medical insurance, the self-pay ratio of medical insurance(amount paid by the patient/total cost for medical services), monthly family income, numbers of public disruptions, self-injury, and suicidal behavior, risk assessment (Level 5: Violence against others, arson, explosion, etc.; Level 4: Repeated smashing behavior, regardless of the occasion, against property or people and cannot be persuaded to stop (including suicide). Level 3: Smashing behavior, regardless of the occasion, against property and cannot be persuaded to stop. Level 2: Smashing behavior against property, confined to the home, and can be persuaded to stop. Level 1: Verbal threats and shouting without hitting. Level 0: None of the above behavior.) This study collected the education level, family history of mental disorders, gender, and demographics from all 367 individuals with SZ.

### Participants

The participants of this study were from a larger epidemiological survey study of severe major mental disorders in Hunan province. The original study used stratified, clustered, and random multistage sampling with a sample size of 72,999 community participants aged 15 years and over from 123 counties and districts within the Hunan province^25^.

The informed consent of the participants or his/her guardian were obtained in both stages of the study. The consent will be signed by the guardian under following conditions: (1) The participants have mental disorder or suspected mental disorder. (2) Participants were under the age of 18. (3) Participants were illiterate persons. All study was approved by the Ethics Committee of the Brain Hospital Hunan Province (the Second People’s Hospital of Hunan Province) and was conducted in accordance with the relevant guidelines. The privacy of the interviewees is carefully considered and protected.

### Quality control

The initial screening stage was conducted by 490 experts who underwent three days of training on the content of GHQ-12 before starting the survey, and the related knowledge was tested at the end of the training. The second phase of the survey was conducted among 200 psychiatrists who were trained in SCID-I/P. Two experts discussed and decided the final diagnosis when the case was difficult.

### Statistical analyses

Frequency analysis of help-seeking behavior, the reasons for non-help-seeking behavior, and the first choice of those seeking help were conducted using IBM SPSS statistics. We also analyzed the impacts of gender and education level on the first choice of help using correlation testing. We analyzed the relationships between help-seeking behavior and gender, family history of mental disorders, monthly family income, whether medically insured, the ratio of self-payment for publicly funded healthcare (self-pay ratio), trouble-making publicly disruptive behavior, self-injury, suicide attempt, risk assessment, urban or rural residence, and education level in the 367 SZ individuals, using the chi-square test or Fisher's exact test. In consideration of the potential interaction between various influencing factors, we conducted a stepwise logistic regression analysis to study each factor’s impact on help-seeking behavior. We included all factors with p < 0.1 (univariate binary logistic regression test) in the multiple logistic regression model. The factors (p < 0.05) in the model were considered to be the most influential factors of help-seeking behavior.

### Ethics approval and consent to participate

This study is approved by ‘the ethics committee of the Brain Hospital of Hunan Province’, the committee’s reference number is ‘Z2019045’. And written informed consent was obtained from a parent or guardian for participants under 16 years old.

## Results

There were 539 individuals with SZ screened from the epidemiological study mentioned above, and 367 individuals who finished the current study questionnaires were recruited into this study.

1. Seeking help versus not seeking help, and influential factors
  1.1. Statistical description of help-seeking behavior
    Two-hundred and fifty-three out of a population of 367 (68.9%) individuals with SZ sought help, while 31.1% did not.
  1.2. The influential factors for help-seeking behavior
    Crosstabs, an SPSS procedure, demonstrated that one’s family history of mental disorders, self-pay ratio and level of education were significantly related to help seeking-help- or- not behavior in individuals with SZ (χ^2^ = 6.621, P = 0.010; χ^2^ = 12.821, P = 0.005; Fisher exact P = 0.0225).
    After pairwise comparison using the chi-square test or Fisher's exact test, we found that the help-seeking behavior ratio is significantly higher in the 30 to 60% self-pay ratio(self-pay fee/total medical expenditure) group than in the 10 to30% self-pay ratio group (chi-square 8.40, p = 0.00375, respectively), with no significant difference in other self-pay groups. Nevertheless, the help-seeking behavior ratio is significantly lower in the illiterate group than in the groups with primary education, junior high school education, and senior high school education (the chi-square and p-value are 4.4557, 0.0348; 6.118, 0.0134; 11.019, 0.000902; respectively). No significant differences in help-seeking behavior exist between other self-pay and educational level groups. The patient group with a family history of mental disorders tends to have a higher help-seeking behavior ratio (chi-square and p-value are 6.621, 0.010, respectively).
    Similarly, help-seeking versus non-help-seeking behavior is not contingent upon gender, monthly family income, medical insurance, publicly disruptive behavior, self-injury, suicide attempts, risk assessment, or urban–rural residence (Table1).
    In consideration of potential interactions between various influencing factors, we used stepwise logistic regression analysis to study each factor’s impact on help-seeking behavior. We included all the p < 0.1 (univariate binary logistic regression test) factors, which included family history of mental disorders, monthly family income, and education level in the model. The result shows that family history of mental disorders is significantly related to whether or not the individual with SZ seeks help (Table1).
2. Barriers to seeking help
  We found that 31.1% of individuals with SZ from the study population did not seek help. Their reasons are as follows (Fig. 1).

**Figure 1:**
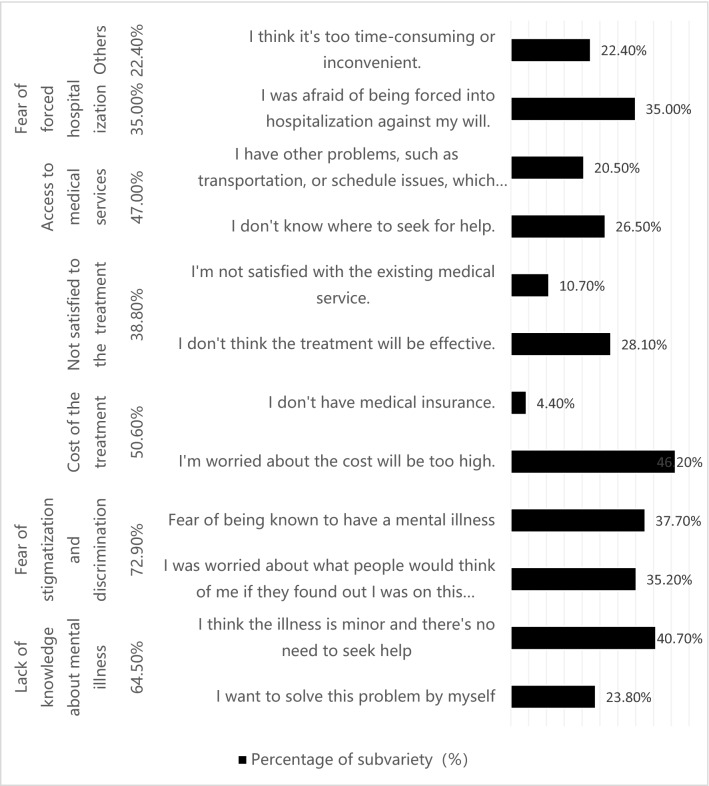
Reasons for non-help seeking behavior.
3. First choices of help-seeking behavior
  3.1 Statistical description of the first choice of help
    Two-hundred and fifty-three out of 367 (68.9%) individuals with SZ in our study did seek help; for 64.6% (n = 163) of those, the first choice was “professional psychiatric service”, with only 41.1% (n = 104) of those admitted to inpatient care. Of the remainder of those seeking help, 30.8% choose non-medical options, in which 18.2% (n = 46) first asked relatives for help (Fig. 2).

**Figure 2:**
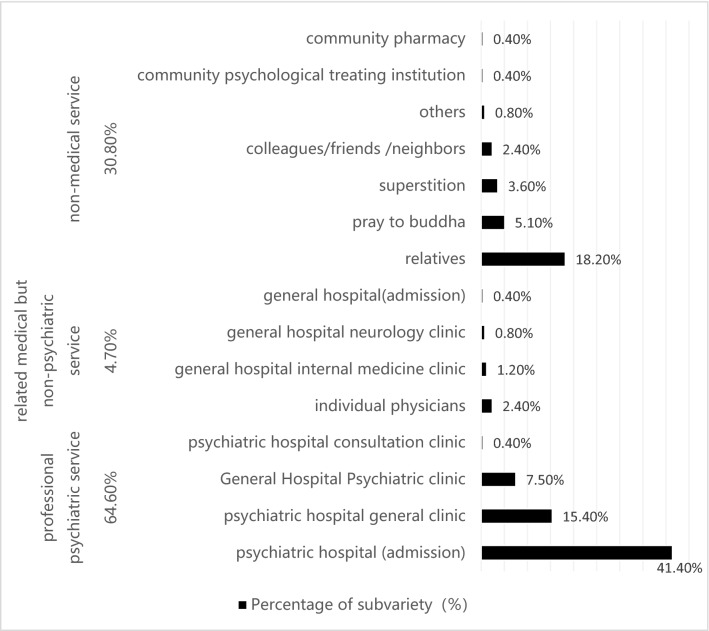
Frequency of the first choice of help for individuals with SZ.
  3.2 The impact of gender and education level on the first choice of help
    The first choice of help is significantly related to education level but not significantly related to gender in the 253 individuals with SZ using the Correlation Test (the P values are 0.007; 0.853; respectively).

**Table 1:** Comparison in help-seeking versus non-help-seeking behavior based on influencing factors.

Influencing factor	Number	Yes	No	Chi-square	P value	Logistic P	Multifactorial logistic P
**Gender**							
Male	180	118	62	1.887	0.17	0.17	
Female	187	135	52				
**Family history of mental disorders**							
Yes	31	29	2	6.621	0.01*	0.021^#^	0.013*
No	189	136	53				
**Monthly family income**							
< 1000 ¥	137	90	47	6.351	0.096	0.078^#^	0.695
1000–3000 ¥	151	100	51				
3000–5000 ¥	62	51	11				
> 5000 ¥	17	12	5				
**Publicly funded healthcare**							
Yes	362	250	112		0.648	0.666	
No	5	3	2				
**Self-pay ratio**							
< 10%	39	31	8	12.821	0.005*	0.161	
10–30%	231	144	87				
30–60%	75	61	14				
> 60%	22	17	5				
**Number of public disruptions**							
Yes	37	21	16	2.087	0.149	0.15	
No	187	129	58				
**Self-injury**							
Yes	4	4	0		0.307	0.999	
No	215	144	71				
**Suicide attempt**							
Yes	4	3	1		1	0.746	
No	214	144	70				
**Risk assessment level**							
F	3	1	2		0.0787	0.13	
E	11	5	6				
D	40	22	18				
C	64	43	21				
B	96	72	24				
A	152	109	43				
**Urban or rural residence**							
Urban	170	118	52	0.033	0.855	0.855	
Rural	197	135	62				
**Educational level**							
Illiterate	50	25	25		0.0225*	0.038^#^	0.095
Primary	115	79	36				
Junior high school	134	95	39				
Senior high school	41	35	6				
Secondary school	14	10	4				
Post-secondary school	5	4	1				
Undergraduate or above	8	5	3				

^#^P < 0.1, *P < 0.05.

*F* Violence against others, arson, explosion, etc., *E* Repeated smashing behavior, regardless of the occasion, against property or people and cannot be persuaded to stop (including suicide), *D* Smashing behavior, regardless of the occasion, against property and cannot be persuaded to stop, *C* Smashing behavior against property, confined to the home, and can be persuaded to stop, *B* Verbal threats and shouting without hitting, *A* None of the above behavior.

## Discussion

1. Key factor determining help-seeking behavior in individuals with schizophrenia
  We identified several key influential factors that determine whether an individual with SZ does or does not seek help.
  1.1. A Family history of mental disorders are significantly related to help-seeking behavior. Furthermore, the first choice of help is significantly related to education level, with those of greater education more likely to receive professional psychiatric services
    A family history of mental disorders is strongly associated with seeking help. Yet, the relationship between a family history of mental disorders and help-seeking behavior of individuals with SZ has not been studied before. As far as we know, this is the first study which investigated family history of mental disorders’ impact on help-seeking behavior of patients with SZ. We considered it a reasonable conclusion basing on our clinical experiences and common sense. Persons and their families with family history of mental disorder had more chances to acquire knowledge about mental disorders (including clinical manifestition, treatment outcome, influence on daily life, etc.) before they got mental disorders. So when they show abnormal behavior or feelings, they and their families had more possibilities to view them as symptoms of certain mental disorders and seek help after that.
    Part of this correlation is that those with a higher level of education tend to have more readily available professional services. Zafar and colleagues found education level to be significantly associated with help-seeking behavior^26^. Those with higher education may have a greater mental health literacy and an improved recognition of mental disorders^27,28^. Our findings indicate that whether individuals with SZ seek professional help depends primarily on how much they know about the disease and their ability to learn more about it.
    This research also found that help-seeking behavior is significantly higher in the 30% to 60% self-pay ratio group than in the 10 to 30% group. This implies that the more people pay themselves, the more likely they are to seek help. This phenomenon may be associated with the special medical insurance model in China. Citizens in China receive greater reimbursement from the government when they choose to receive medical services from hospitals near their residence (for example: if a woman lives in a rural county, she would need to pay only 10 to 13% of the cost of treatment at a rural hospital within the same county; whereas, she would need to pay from 30 to 60% of the cost if she went to an urban hospital, and nearly 70% of the cost to be treated further within the province or in another province. This may indicate that people who have more money for healthcare may pay more attention to their health, and maybe those with more money are more medically literate. Which leads to higher help-seeking willingness?
  1.2. There were no significant differences in whether or not individuals sought help based on gender, monthly family income, having medical insurance, public disruptions, self-injury, suicide attempts, risk assessment, or urban versus rural residence
    Gender, monthly family income, possessing medical insurance, publicly disruptive behavior, self-injury, suicide attempt, and risk assessment, urban versus rural residence did not correlate to help-seeking behavior. These negative findings are inconsistent with some previous studies that find patients’ gender having a close relationship to whether or not patients seeking help. They attribute the correlation of gender to help-seeking behavior to differences between the sexes in causal attribution of their illnesses^29,30^. Karam and colleague’s study shows financial concerns as a common barrier for people with mental illness seeking treatment^31^. Kilany (2018) found that healthcare utilization by individuals with severe mental illness influences by medical insurance and urban–rural dispersal^32^. Huang and colleague’s 2019 study finds that an individual’s perception of a higher severity level indicates greater impetus for treatment^33^. The sample size of our study is not big enough, so we assume that the numbers of public disruptions, self-injury, suicide attempt, and risk degree assessment are few and may not be represented in this small sample. Factors including gender, monthly family income, and medical insurance, residential did not emerge as related to whether individuals seek help, which may have something to do with the sample source. Uniquely, our population is derived from a community sample, whereas previous studies simply used hospitalized populations. The population of individuals with SZ within the larger community differs from a hospitalized population in the severity of the disease state, with the latter typically more severe than the general population. These populations are not homogeneous.
  1.3. A disconcerting number of individuals who can be diagnosed with schizophrenia among the general population do not seek help due to common barriers
    Our study reveals a distressing statistic, that nearly one-third of individuals in the general population who can be diagnosed with SZ do not seek help. Similarly, a study by Zhang and colleagues (2013) found that 28.1% of patients with mental illness in the urban areas of northern China do not seek any form of help either^34^. These results contradict a study in the rural community of Liuyang, China, which showed far fewer than half of the larger percentages of individuals with SZ not seeking help (only 12.7%)^35^. This stark contrast may be due to differences in sample sources and study procedures.
    In our study, the main reasons for not seeking help include fears of stigmatization and discrimination (72.9%), poor mental health literacy (64.5%), concerns over the cost of the treatment (50.6%), and limited access to medical services (47.0%). Research from Mavrogiorgou (2015) shows that roughly 40% of their study participants reported fears of stigmatization and discrimination as major barriers to help-seeking behavior^36^. Other research also found stigmatization as a serious impediment to obtaining help^37^. Furthermore, Ho and colleagues (2008) pointed to the lack of knowledge about the available treatment, the inability to afford the high cost of treatment, and lack of transportation as the pivotal barriers^38^. One study of individuals in Ningxia, China found that a general lack of mental health literacy and limited access to specialized medical institutions were the predominant reasons that individuals with severe mental illness chose non-specialized hospitals or did not seek medical treatment^39^. The exact same barriers predominated in our study as well, which indicates that to a large degree social factors dictate help-seeking behavior. Increased social tolerance and acceptance of SZ, improved general awareness of SZ, and strengthened access to professional psychiatric services are needed.
2. First choices for help-seeking behavior
  2.1 Professional psychiatric services and non-medical options are the preferred resource for individuals with SZ who seek help, with over twice as many choosing professional psychiatric services (64.6%, n = 163) over non-medical options (30.8%, n = 78). The top three choices of non-medical options include relatives, praying to Buddha, and traditional and/or faith healers. This study found psychiatric hospitals and relatives to be the most common first choices of individuals with SZ seeking help, whereas some studies show psychiatrists and psychiatric hospitals as the most common first choices^16^. Nonetheless, other studies show general medical hospitals and physicians as the first choice^10,20^. Such disparity in findings may arise from the different medical structures and low accessibility of psychiatrists and mental hospitals. Their results also contradict ours in that general medical support and hospitalization was the most common first choice. Some studies also indicate that religion^15^ and traditional/faith healers^12^ are the most common first choices. However, our study showed the help-seeking behavior of individuals with SZ in the Hunan province of China is comparatively more scientific. In terms of relatives being an initial means of support, a previous study in southern India states that 16.8% of late adolescents with SZ seek help from their mothers^13^, while a study from Nigeria declares that 91% of individuals’ first contact of treatment is initiated by relatives^15^. This research indicates that relatives play a very important role in the help-seeking behavior of individuals with SZ in Hunan province with family support also being positive and strong in China. Research also indicates that improving basic mental health literacy can expand the utilization of mental health services^40^.

### Novel study design using community-based sampling

We believe this is the first study that has investigated help-seeking behavior in individuals with SZ using a community sample at a provincial level. It is of great significance for public health, because it offers information about the current situation about seeking help behavior of individuals with SZ in China, and indicates the future direction of medical education development. The main limitation of this study was as follows: 1) There are too few investigators available for this study, we could not personally interview all of the individuals suspected of having SZ who were screened in the first stage; therefore, a considerable proportion of individuals with SZ may have been missed.2)Our study is a small part of a larger epidemiological study of severe mental illness in the Hunan province, missing values did result in the tremendous workload at hand.

## Conclusions

In conclusion, we found that about one-third of individuals with SZ in Hunan province do not seek help. The primary reasons for this large percentage include fear of stigmatization, poor mental health literacy, concerns over cost, and limited access to medical services. Individuals who do seek help tend to choose psychiatric hospitals and relatives as their first choice of help. A family history of mental disorders was significantly associated with help-seeking behavior. The first choice of help correlates significantly to education level; correspondingly, those with more education are more likely to access professional services. Our findings indicate mental health literacy is meaningful and important not only to individuals with mental disorders but to their relatives as well. It also indicates that mental health literacy should be augmented in populations with less education and populations without a family history of mental disorders. Our study also indicates that an increased tolerance and acceptance of SZ within society and improved access to professional medical services should be encouraged in the future.

## Data Availability

The datasets used and/or analyzed during the current study are available from the corresponding author on reasonable request.

## Abbreviation

SZ: Schizophrenia
